# Live Patient Encounters: A Perspective From Second-Year Medical Students

**DOI:** 10.7759/cureus.46274

**Published:** 2023-09-30

**Authors:** Emily R Littman, Shazia Beg

**Affiliations:** 1 Medicine, University of Central Florida College of Medicine, Orlando, USA; 2 Rheumatology, University of Central Florida College of Medicine, Orlando, USA

**Keywords:** medical student performance evaluation, medical student training, medical student perspective, teaching strategies, medical education curriculum

## Abstract

Background

Interactive patient cases have been shown to be a valuable resource in medical education. Previous studies have demonstrated that using patients as teachers can help students improve clinical reasoning and have educational benefits; however, there is limited research on student feedback on patients as teachers. The objective of this study is to evaluate second-year medical students' (MS2s) perceptions of patient encounters during the teaching of the Skin and Musculoskeletal System Course (BMS 6635).

Methods

A retrospective descriptive study on prospectively maintained survey data was performed. Following course completion, MS2s were surveyed on their experience from four to five live patient encounters at the University of Central Florida College of Medicine from 2016-2022. The interactive cases involved patients with dermatologic, autoimmune, and musculoskeletal diseases. All MS2s enrolled in BMS 6635 were included. Statistical analysis was performed on survey responses to students' perceptions of live patient encounters.

Results

Seven hundred surveys were completed following the interactive patient encounters. Ninety percent of participants answered that they enjoyed the cases, 92% agreed the cases were an appropriate learning experience for their education, and 76% agreed the cases helped with material retention. From 2016 to 2022, there was a slight decrease in enjoyment in the cases over time (97%, 88%, 93%, 94%, 86%, 81%, p<.001, respectively), and student agreement that patient cases were an appropriate learning experience in their education (98%, 92%, 94%, 95%, 93%, 84%, p=.001, respectively), but overall remained greater than 80% satisfaction.

Conclusions

Patient cases are perceived to be a valuable educational resource by second-year medical students and therefore should be integrated in medical curricula. Students enjoyed patient cases, believed they had an educational benefit, and perceived they aided in material retention.

## Introduction

Patients have increasingly been used as teachers in medical education [1]. This approach has garnered positive feedback from students and is demonstrably equivalent in quality to that which is provided by medical educators [2-5]. Using standardized patients to teach physical exam skills has been a valuable component of medical school curricula for a while, as it teaches students empathy, comprehensive understanding, and cultural sensitivity [6]. Studies have found that with proper training, patients can help improve the students' physical examination skills, as well as students' communication and confidence [1]. Even though patient teaching in areas such as physical examination skills has been shown to be an integral part of medical education, there is limited research on patients as medical educators in the classroom [6].

The use of patients in the classroom provides a unique perspective to students and allows them to build knowledge in addition to skills. Since clinical presentation varies along the continuum of chronic disease, experiential learning introduces opportunities to students that may not otherwise be obtained in a traditional classroom lecture [1]. Furthermore, it has been shown that the personal connection brought about by using patients as experts facilitates enjoyment and engagement among students. A study by Wykurz et al. demonstrated that 59% of students found patient teachers to be more enjoyable than professors as a result of the more personal accounts [1]. Patients, too, have found teaching medical students to be an enjoyable way to contribute to the knowledge of a generation of future physicians by sharing their experiences [1]. Some studies have shown that patients feel more empowered and informed through their participation as a patient in a learning environment [7,8].

Studies are, however, limited on students' perspectives of patients conveying personal experiences with chronic disease and instead consist mostly of patients teaching physical examination skills [1,5,9,10]. Thus, policy documents from medical boards such as the Association of American Medical Colleges have recommended that medical schools create opportunities for early patient contact so students can have a better understanding of patients' disease experiences [9,11]. Our institution follows the traditional medical school curriculum in which students' first two years of medical school are non-clinical years with little patient interaction, and the last two years of school are clinical training.

Although a few studies have shown that using patients as teachers helps students develop clinical reasoning, motivates students to learn, and has important educational benefits, there is little research understanding medical student feedback during these interactive patient cases. The objective of this study is to evaluate second-year medical students' (MS2s) perceptions of interactive patient encounters during the teaching of dermatologic and rheumatologic conditions in the Skin and Musculoskeletal System course based on surveys collected from 2016-2022.

This article was previously presented as a poster at the 2022 American Medical Association (AMA) Research Challenge on October 21, 2022, and as a poster presentation at the Congress of Clinical Rheumatology, San Diego, California, on October 20, 2022.

## Materials and methods

Live patient encounter

The live patient encounter consisted of a three-hour block in which students interacted with four to five patients with chronic diseases during this time. Patient diagnoses included systemic lupus erythematosus, psoriatic arthritis, psoriasis, dermatomyositis, scleroderma, ankylosing spondylitis, osteoarthritis, and pyoderma gangrenosum. Each live patient encounter consisted of about 30-45 minutes of student-patient discussion regarding symptoms, diagnosis, treatment, and overall patient perspectives and was moderated by three faculty members teaching the course. The live patient encounter sessions began with the patient talking about his/her initial symptoms of their disease presentation. Images of rashes, X-rays, physical exam findings, and lab findings were then shown to the class. Students were given the opportunity to ask the patient any other questions they wanted to know about the history to help them come up with a diagnosis. Differential diagnosis and treatment plan was discussed for each patient. At the end of each patient session, there was an open forum for any additional comments/questions for the patient. All students saw the same four to five patients for each live patient encounter.

The format of these sessions was modified as a necessity of the COVID-19 pandemic with changes in learning platforms from in-person to virtual learning. Prior to the COVID-19 pandemic (academic years 2016-2020), live patient encounters consisted of students being placed into groups and the patient teachers rotating between student groups so that each group met with all of the patients. In the first academic year of the COVID-19 pandemic (academic year 2020-2021), the curriculum was converted to a completely virtual format to ensure patient, student, and faculty safety. The session consisted of both the students and patients interacting on an online virtual platform. The next year (academic year 2021-2022), the session was converted to a hybrid format, which consisted of the students in-person in a classroom together with the patients in an online virtual format to limit their COVID-19 exposure due to their underlying conditions. During the virtual and hybrid format, the entire class was interacting with the same patient at the same time.

Sample

This is a single-center retrospective descriptive study utilizing a sample of MS2s enrolled in a skin and musculoskeletal system course (BMS 6635) at the University of Central Florida College of Medicine from 2016 to 2022. An online survey was distributed via email to students upon the completion of the course. This survey was distributed at the same time as the mandatory end-of-course evaluations. It was sent as a separate survey, and completion was voluntary. From 2016 to 2022, there were a total of 700 students enrolled in the course who received the survey. MS2s enrolled in BMS 6635 from 2016-2022 were included.

Survey

We surveyed MS2s to assess their experience from four to five interactive patient cases at the University of Central Florida College of Medicine during the academic years 2016-2022. The survey ranged from three to five questions due to adaptations of the surveys throughout the years (Table 1). Question number five pertained to only the two academic years that were affected during the COVID-19 pandemic and was not a focus of this study; therefore, this question was not included in this study. The survey consisted of a five-point Likert scale ranging from strongly agree, agree, neutral, disagree, and strongly disagree. The survey was designed to assess students' engagement and satisfaction in live patient encounters throughout the years 2016 to 2022.

**Table 1:** Change in survey questions asked to medical students from 2016 to 2022

Questions asked in survey	2016-2017	2017-2018	2018-2019	2019-2020	2020-2021	2021-2022
Question #1: I enjoyed the live patient cases	x	x	x	x	x	x
Question #2: The live patient cases were an appropriate learning experience at this stage in my education	x	x	x	x	x	x
Question #3: We should increase the number of patients in the live patient cases				x	x	x
Question #4: The live patient cases helped me remember the diseases well for the exam				x	x	x

Statistical analysis

Survey responses on the five-point Likert Scale were re-classified and dichotomized to "agree" for the responses "agree" and "strongly agree" and to "disagree" for the responses "neutral", "disagree", and "strongly disagree". Chi-squared (χ2) testing was performed on survey responses obtained from 2016 to 2022. Linear regression analysis was performed on significantly different survey responses. Statistical analysis was performed using MathWorks MATLAB_R2020a (MathWorks, Natick, Massachusetts). Significance was defined as p<0.05.

## Results

Demographics

Seven hundred surveys were completed at the conclusion of the study period. This consisted of 116, 116, 126, 115, 106, and 121 participants during the academic years 2016-17, 2017-18, 2018-19, 2019-20, 2020-21, and 2021-2022, respectively. Respondents were all MS2s enrolled in a skin and musculoskeletal system course (BMS 6635) at the University of Central Florida College of Medicine from 2016 to 2022.

Student Enjoyment

**Figure 1:**
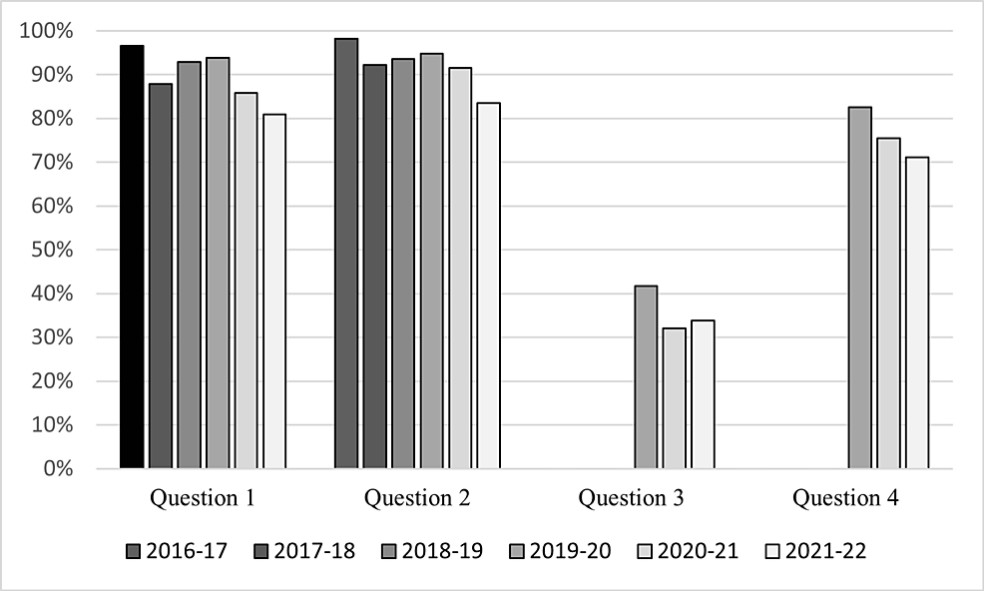
Agreement trend in medical students' response to survey questions from 2016 to 2022

Ninety percent of survey participants answered that they enjoyed the interactive patient cases (Table 2). A significant difference was noted in student enjoyment throughout the years studied (97%, 88%, 93%, 94%, 86%, 81%, p<.001, respectively per year) (Figure 1, Table 3). Linear regression analysis demonstrated a significant decrease in the percentage of student enjoyment in live patient encounters from 2016-2022 (β=-0.02, SE=0.01, p<.001) (Beta, Standard Error, *p*-value; Table 4).

**Table 2:** Medical student survey responses of agreement to live patient cases comparing those who agree to those who disagree

Questions	Student responses			
	Agree n (%)		Disagree n (%)	Total
I enjoyed the live patient cases		628 (90%)	72 (10%)	700
The live patient cases were an appropriate learning experience at this stage in my education		646 (92%)	54 (8%)	700
We should increase the number of patients in the live patient cases		123 (36%)	219 (64%)	342
The live patient cases helped me remember the diseases well for the exam		261 (76%)	81 (24%)	342

**Table 3:** Medical students' change in survey responses of agreement over time from 2016 to 2022 * = statistical significance defined as p<0.05.

Questions	2016-2017	2017-2018	2018-2019	2019-2020	2020-2021	2021-2022	p-value
I enjoyed the live patient cases	97%	88%	93%	94%	86%	81%	< .001*
The live patient cases were an appropriate learning experience at this stage in my education	98%	92%	94%	95%	93%	84%	.001*
We should increase the number of patients in the live patient cases	-	-	-	42%	33%	34%	.274
The live patient cases helped me remember the diseases well for the exam	-	-	-	83%	77%	72%	.111

**Table 4:** Linear regression analysis on medical students' survey response agreement from 2016-2022 * = statistical significance defined as p<0.05.

Questions		Beta	SE	p-value
I enjoyed the live patient cases		-0.02	0.01	< .001*
The live patient cases were an appropriate learning experience at this stage in my education		-0.02	0.01	.001*
We should increase the number of patients in the live patient cases		-0.04	0.03	.194
The live patient cases helped me remember the diseases well for the exam		-0.06	-2.00	.047*

Student learning experience

Ninety-two percent of students agreed that the interactive patient cases were an appropriate learning experience for their education (Table 2). There was a significant difference noted in students' belief that the interactive patient cases are a valuable learning experience in their education (98%, 92%, 94%, 95%, 93%, 84%, p=.001, respectively per year; Figure 1, Table 3). Linear regression analysis demonstrated a significant decrease from 2016 to 2022 in medical students agreeing that live patient encounters are a valuable learning experience (β=-0.02, SE=0.01, p=.001; Table 4).

Number of patients for interactive cases

Only 36% agreed that the number of patients in the interactive patient cases should be increased (Table 2). While not significant, throughout the years, there was a difference in students believing that there should be more patients in the live patient encounters (42%, 33%, 34%, p=.274; Figure 1, Table 3). Linear regression analysis demonstrated a decrease from 2019 to 2022 in students agreeing that there should be a greater number of patients in the interactive cases (β=-0.04, SE=0.03,p=.194; Table 4).

Student memory retention

Seventy-six percent of students agreed that the patient encounters helped them retain knowledge of the disease processes (Table 2). While not significant from 2019 to 2022, there was a clinical difference in students agreeing that the live patient cases helped with memory retention for the exam (83%, 77%, 72%, p=.111; Figure 1, Table 3). Linear regression analysis from 2019-2022 demonstrated a significant decrease in students' perception that the cases helped with memory retention (β=-0.06, SE=-2.00, p=.047; Table 4).

## Discussion

It has been well documented that interactive patient cases are a valuable resource in medical education and should be integrated into medical curricula [1,12]. Prior studies mostly consist of patients teaching physical examination skills rather than the effects of chronic disease and are limited to the students' experience with patients as teachers. By using survey data over six years to assess students' engagement and enjoyment in live patient encounters, this study demonstrates the impact of live patient encounters on students' learning experiences and enjoyment of course material. We believe that these encounters should have a place in the medical school curriculum, such as in our medical school's curriculum for the Skin and Musculoskeletal Systems module.

From our study, we found that students generally enjoyed this experience and would like to continue live patient encounters, with 90% of medical students enjoying the interactive patient cases, 92% agreeing that the interactive patient cases were an appropriate learning experience for their education, and 76% agreeing that the patient encounters helped them retain knowledge of the disease process. Our results build upon previous studies, which have shown that using patients as teachers to be an enjoyable and meaningful approach to learning [1]. Our findings support the assertions by Philpotts et al. and Jha et al. that providing students with personalized insight complements the theory taught by academics to aid in the understanding of concepts and retention of knowledge [3,13].

Most students (64%) disagreed that the number of patients in the interactive patient cases should be increased. While student reasoning for their answers to this question was not explored in this study, we hypothesize that the negative responses could be due to the changes in delivery of the interactive cases over the years. In some years, having four or five patients in a three-hour block did not give sufficient time for students to interact with each patient. It is unclear from our results as to whether the number of cases presented was adequate or if a smaller number would be beneficial, which could further be investigated in future studies.

While our results showed overall positive feedback from the patient encounters, it should be noted that over the six-year period from 2016 to 2022, there was a slight significant decrease in positive feedback regarding this approach. This may be due to a few adaptations of the interactive patient encounters over the years. Some of the adaptations included small group sessions and entire class sessions, and the last two years were virtual sessions due to the COVID-19 pandemic. Another possible reason could be attributed to technological advancements and the increased use of third-party resources among students [14,15]. With recent increases in technology, studies have found that medical students have increasingly used third-party resources to supplement formal resources provided by their schools [16]. These third-party resources have become popular in medical school education, such as professional third-party tutors, question banks, memory resources, and other medical resources [14-17]. This may be contributing to the decline seen in our results. This decline, while small, is an important consideration for academic institutions.

It is well documented that doctors in practice think that their practice and clinical decision making is enhanced by ongoing learning and engagement with their patients [1]. We hope this study will have a similar effect on medical students. We found these cases to be a beneficial addition to our medical school curriculum, as indicated by our students' positive feedback on these patient encounters in memory retention, student enjoyment, and learning experience. While this is a single-center study, our experience has been positive in the implementation of live patient encounters, and we hope for this to be a model for other institutions to implement in their medical education programs.

Limitations

While we believe this to be an important study, there are notable limitations to be identified. This is a single institutional study focusing on a single course in medical education. Our study included a non-validated survey that underwent multiple adaptations during the study period. Additionally, during the last two years, we had to adjust the live patient encounters to a virtual format due to the COVID-19 pandemic, which may have affected the survey results on participant satisfaction over the years. This change in format and satisfaction may warrant further investigation into the different delivery modalities of live patient encounters.

## Conclusions

Overall, students enjoyed the interactive patient cases, believed they were an appropriate addition to their medical education even with a minimal number of cases, and perceived they aided in retention of material. Live patient encounters in the pre-clinical years may be considered for wider inclusion in the medical curricula. However, further research is warranted into the delivery format and optimization of these live patient encounters.
